# Large Lateral Photovoltaic Effect in Metal-(Oxide-) Semiconductor Structures

**DOI:** 10.3390/s101110155

**Published:** 2010-11-11

**Authors:** Chongqi Yu, Hui Wang

**Affiliations:** Department of Physics, The State Key Laboratory on Fiber Optic Local Area Communication Networks and Advanced Optical Communication Systems, Key Laboratory for Thin Film and Microfabrication Technology of the Ministry of Education, Shanghai Jiao Tong University, 800 Dongchuan Road, Shanghai 200240, China

**Keywords:** lateral phtotvoltaic effect (LPE), metal-semiconductor (MS), metal-oxide-semiconductor (MOS), PACS 72.40.+w, 73.40.Ns, 73.40.Qv

## Abstract

The lateral photovoltaic effect (LPE) can be used in position-sensitive detectors to detect very small displacements due to its output of lateral photovoltage changing linearly with light spot position. In this review, we will summarize some of our recent works regarding LPE in metal-semiconductor and metal-oxide-semiconductor structures, and give a theoretical model of LPE in these two structures.

## 1. Introduction

The lateral photovoltaic effect (LPE) is an attributive character of some semiconductor structures. Since the LPE effect was first discovered by Schottky [1] and later in 1957 expanded upon by Wallmark in floating Ge p-n junctions [2], it was boosted very quickly in many different semiconductor systems, such as Ti/Si amorphous superlattices [3–6], modulation-doped AlGaAs/GaAs heterostructures [7], hydrogenated amorphous silicon Schottky barrier structures [8–13], organic semiconducting polymers [14,15], perovskite materials [16–19], and two-dimensional electron systems (2DES) [20]. Noticeable advances were achieved also by fabricating these devices on flexible substrates [21], such as a heterojunction of amorphous silicon (a-Si:H)/ZnO:Al.

Due to the fact its output of lateral photovoltage (LPV) changes linearly with light spot position, this effect can be used in position-sensitive detectors (PSDs) which can detect very small displacements [22–35]. The main area of application of PSDs is in precision optical alignment, such as biomedical applications, robotics, process control, medical instrumentation, and position information systems [36–39]. Other attractive applications include surface profiling, rotation monitoring, telephone information systems, angle measurements, triangulation-based distance sensors, guidance systems and roles where precise automated control is required [40–43]. PSDs based on the LPE can provide continuous optical information over large areas with no internal discontinuities, which is the major advantage over arrayed discrete devices such as charge coupled devices and photodiodes.

In judging whether a particular device works well, the two main criteria are the linearity and the sensitivity. Early works concerning LPE only mentioned their LPV sensitivities of less than 10 mV/mm [3–5], and later many works with larger LPV sensitivities have been reported. For example, Jin’s and Lu’s group have reported their results of 10–60 mV/mm of LPV sensitivities in perovskite-based p-n junctions [16–19], and Henry’s group have reported their results of 5–25 mV/mm of LPV sensitivities in Schottky barrier structures [8–12].

**Figure 1.**
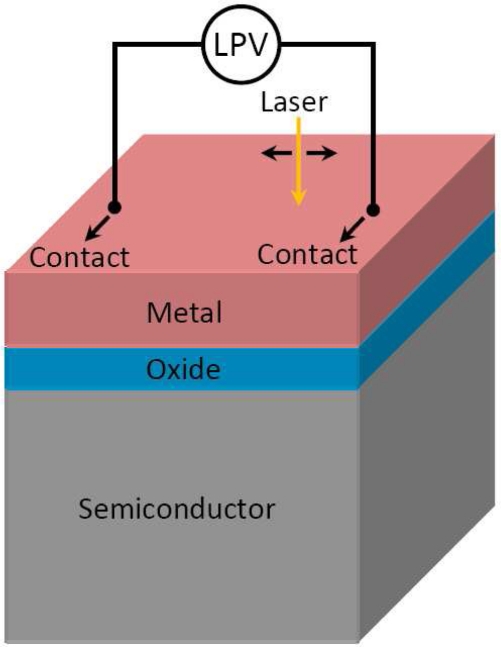
Diagram of LPV measurement in a MOS structure on metal side.

In this paper, we will give a brief review about our recent works which deal with LPE in metal-semiconductor (MS) and metal-oxide-semiconductor (MOS) structures [44–57] (Figure 1 shows the device structure), including a theoretical explanation of LPE in these structures. In fact, MS and MOS structures are versatile materials and have been treated as solar cells for many decades [58–63], but they serve as LPE materials is pretty new, in particular their large LPE can be directly obtained or measured on the metal side.

## 2. Experimental Results

### 2.1. Fabrication and Measurement

**Figure 2.**
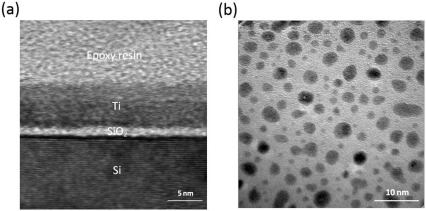
**(a)** A cross-section TEM image of the calibration MOS structure of Ti(6.2 nm)/SiO_2_(1.2 nm)/Si. **(b)** TEM planar-view image of the top polycrystalline Ti film of the structure.

All the metal films and oxide layers with different thickness were fabricated on n-type single crystal Si (111) substrates at room temperature by dc magnetron reactive sputtering. The thickness of the wafers is around 0.3 mm and the resistivity of the wafers is in the range of 50–80□Ωcm at room temperature. The Si substrate was covered with a thin native 1.2 nm SiO_2_ layer that was been confirmed by high-resolution transmission electron microscopy (TEM), as shown in Figure 2(a). The base pressure of the vacuum system prior to deposition was better than 5.0 × 10^−5^ Pa. Many high purity metal targets were used. The deposition rates (or growth rates), determined by a stylus profile meter on thick calibration samples, were mostly less than 0.5 Ås^−1^. All the thicknesses of fabricated films were determined by calculating deposition time multiplying deposition rates. The low deposition rate ensures the accuracy of determining film thickness, even for a very thin film. Figure 2 shows a TEM image of one of our samples of Ti(6.2 nm)/SiO_2_(1.2 nm)/Si by sputtering.

The samples were cut into rectangles and scanned with a laser focused into a roughly 50 μm diameter spot at the surface and without any spurious illumination (e.g., background light) reaching the samples. All the contacts (less than 1 mm in diameter) to the films were formed by alloying indium and showed no measurable rectifying behaviour. Experimental details are similar with our recently published papers [46–48].

### 2.2. LPE in MS Structure

**Figure 3.**
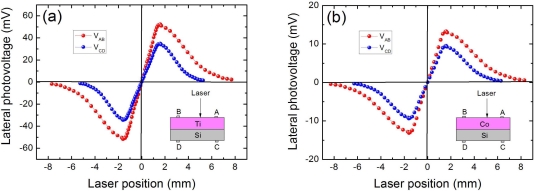
**(a)** LPV measurement on both metal and semiconductor sides in MS structure of Ti(6.2 nm)/Si. **(b)** LPV measurement on both metal and semiconductor sides in MS structure of Co(6.2 nm)/Si. Here the contacts’ distance is 3.2 mm, and the laser wavelength and power are 632 nm and 3 mW, respectively.

Figure 3 shows our experimentally observed LPEs in two kinds of MS structures of Ti(6.2 nm)/Si and Co(6.2 nm)/Si (experimentally there is no LPE in Ti or Co metal films deposited on a glass because the metal films give rise to an almost equipotential and they are not photosensitive). To give a better comparison of LPE between them, Ti and Co are prepared with a same thickness. Please note the thin native SiO_2_ layer cover on Si substrate here was removed before fabrication. As shown in Figure 3, both the metal side LPE (*V_AB_*) and the semiconductor side LPE (*V_CD_*) in the two structures are obvious. Since only the linear dependence of LPV between two contacts is useful for PSDs, we concentrate on the results obtained between two contacts for our MS devices in the following discussion. Clearly, we can see from Figure 3 that all the LPVs show a good linear characteristic response to the laser position. The nonlinearity, also known as position-detection error, is defined [64,65] as:

(1) $$\text{Nonlinearity}\equiv \delta \equiv \frac{2\times \text{rms}\;\text{deviation}}{\text{Measured}\;\text{full}\;\text{scale}}$$

According to Equation (1), the nonlinearities of LPVs on the metal side are 4.8% for Ti/Si and 3.9% for Co/Si, and those on the semiconductor side are 8.3% for Ti/Si and 6.2% for Co/Si, respectively. This means the linearity in these MS structures are pretty good.

Another important key for LPE is the sensitivity of LPV response to the laser position. As shown in Figure 3, the LPV sensitivities on the metal side is 40.0 mV/mm for Ti/Si and 31.2 mV/mm for Co/Si, which are relatively higher than the 27.2 mV/mm for Ti/Si and 21.8 mV/mm for Co/Si on the semiconductor side. These results demonstrate that the metal side LPEs are quite obvious, and (in this case) have better LPV sensitivities than those in semiconductor side. We must stress here the LPEs in early studies were mostly observed in semiconductor side, and the metal side LPEs were always negligibly small. This is because the metal side LPE is very sensitive to metal film thickness. If the thickness is outside an appropriate range, the metal side LPE can hardly be observed. Only when the metal film thickness is reduced to several nanometers, then the metal side LPE can become obvious (will be discussed further in Sections 2.4 and 3.5).

Interestingly, with a same metal thickness, Ti/Si structure presents a much higher LPV sensitivity than that in Co/Si structure no matter it is measured on metal side or on semiconductor side. This relates with the properties of metal materials, and will be interpreted in Section 3.7.

### 2.3. LPE in MOS Structure

**Figure 4.**
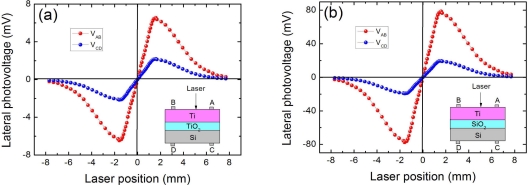
**(a)** LPV measurement on both metal and semiconductor sides in MOS structure of Ti(6.2 nm)/TiO_2_(1.2 nm)/Si. **(b)** LPV measurement on both metal and semiconductor sides in MOS structure of Ti(6.2 nm)/SiO_2_(1.2 nm)/Si. Here the contacts’ distance is 3.2 mm, and the laser wavelength and power are 632 nm and 3 mW respectively.

Figure 4 shows our experimentally observed LPEs in two kinds of MOS structures of Ti(6.2 nm)/TiO_2_(1.2 nm)/Si and Ti(6.2 nm)/SiO_2_(1.2 nm)/Si. Please note the TiO_2_ oxide layer in Ti/TiO_2_/Si structure is fabricated by sputtering and the native SiO_2_ layer cover on Si substrate had been removed before fabrication (the SiO_2_ oxide layer in Ti/SiO_2_/Si structure is native). Obviously, Ti/SiO_2_/Si presents a much higher LPV sensitivity of 48.6 mV/mm in metal side and 12.1 mV/mm in semiconductor side. However, Ti/TiO_2_/Si only presents a relatively low LPV sensitivity of 4.0 mV/mm in metal side and 1.4 mV/mm in semiconductor side. Compared with the LPE in Ti/Si (see Figure 3(a)), the LPE sensitivity in Ti/SiO_2_/Si structure is increased, but reduced in the Ti/TiO_2_/Si structure. This is because for the Ti/TiO_2_/Si structure, the TiO_2_ oxide layer serves as a higher barrier compared with that of Ti/Si, and this barrier will reduce more of laser-excited electrons tunneling from semiconductor to metal, and thus results in a relatively small LPE. However, for Ti/SiO_2_/Si structure, though SiO_2_ oxide layer also serves as a barrier, some interface states will exist within the forbidden band of the semiconductor due to the formation of SiO_2_-Si junction in this MOS structure [66]. This will increase the density of laser-excited electrons, thus results in an enhancement of LPE.

### 2.4. Metal Thickness Effect

**Figure 5.**
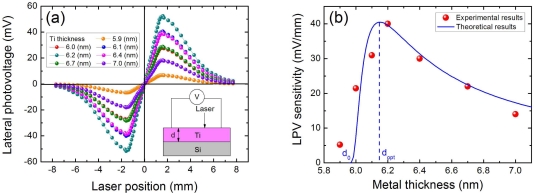
**(a)** LPV measurement in MS structures of Ti/Si with different Ti thickness. **(b)** LPV sensitivities as a function of Ti thickness in Ti/Si structures. Here the contacts’ distance is 3.2 mm, and the laser wavelength and power are 632 nm and 3 mW respectively. Solid line is the plot of Equation (17), where the parameters are chosen as *α_m_* = 5 × 10^6^ and *d*_0_ = 5.95 (nm).

To further investigate the thickness effect of metal film on LPE in MS structure, we measured the LPE with different Ti thickness in Ti/Si structures, as shown in Figure 5(a). We can clearly see from Figure 5(b) that the position sensitivity of LPV in Ti/Si structure will decrease when the thickness of Ti is away from the optimum value of 6.2 nm. This is why we choose the Ti(6.2 nm)/Si as a control sample in **2.2** because, at this thickness, Ti/Si structure has the strongest LPE. Therefore, in order to obtain a large LPV in MS structure, an appropriate metal thickness is crucial. In addition, it is also seen from Figure 5(b) that a threshold thickness of 5.9 nm exists in this thickness effect. Only when the Ti thickness is larger than this threshold value, LPE can occur in Ti/Si structures.

### 2.5. Oxide Thickness Effect

**Figure 6.**
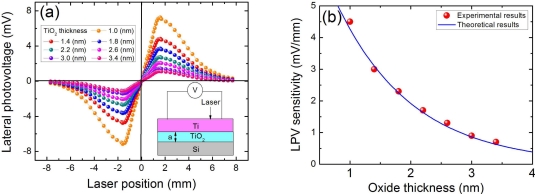
**(a)** LPV measurement in MOS structures of Ti(6.2 nm)/TiO_2_/Si with different TiO_2_ thickness. **(b)** LPV sensitivities as a function of TiO_2_ thickness in Ti(6.2 nm)/TiO_2_/Si structures. Here the contacts’ distance is 3.2 mm, and the laser wavelength and power are 632 nm and 3 mW respectively. Solid line is the plot of Equation (20), where the parameters are chosen as *α*_0_ = 0.3 (nm) and *α*_t_ = 1.25 (nm).

It can be seen from Section 2.3 [Figure 4(a)] that the TiO_2_ oxide layer in Ti/TiO_2_/Si structure will deteriorate the LPE because the TiO_2_ barrier will reduce the electrons tunneling from semiconductor to metal. To further investigate the thickness effect of oxide layer on LPE in MOS structure, we measured the LPE with different TiO_2_ thickness in Ti(6.2 nm)/TiO_2_/Si structures, as shown in Figure 6(a). We can find from Figure 6(b) that the LPV sensitivity will decrease with the increase in oxide thickness. This is because the increase in oxide thickness will increase the barrier’s width, thus lead to the electrons tunneling from semiconductor to metal becoming more difficult.

**Figure 7.**
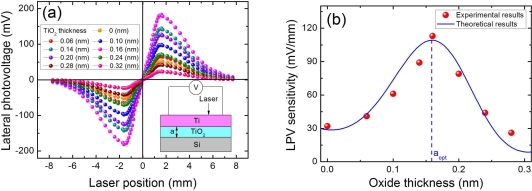
**(a)** LPV measurement in MOS structures of Ti(6.2 nm)/TiO_2_/Si with different super-thin Ti thickness. **(b)** LPV sensitivities as a function of super-thin TiO_2_ thickness in Ti(6.2 nm)/TiO_2_/Si structures. Here the contacts’ distance is 3.2 mm, and the laser wavelength and power are 632 nm and 3 mW respectively. Solid line is the plot of Equation (20), where the parameters are chosen as *α*_0_ = 0.3 (nm) and *α*_t_ = 1.25 (nm).

Interestingly, as this oxide layer is reduced to 0.16 nm, the LPE in Ti(6.2 nm)/TiO_2_/Si structure will be greatly enhanced. As shown in Figure 7(a), the LPV sensitivity in Ti(6.2 nm)/TiO_2_(0.16 nm)/Si structure can attain 113 mV/mm, which is much larger than the 4.0 mV/mm in Ti(6.2 nm)/TiO_2_(1.2 nm)/Si [see Figure 4(a)] and even larger than the 40.0 mV/mm in Ti(6.2 nm)/Si [see Figure 3(a)]. This means the super-thin oxide layer in this situation no longer serves as a high barrier which deteriorates the LPE. To fully investigate this LPE enhancement effect, we measured the LPEs with many different super-thin thicknesses of oxide layer varying from 0.06 nm to 0.32 nm, as shown in Figure 7(a). Figure 7(b) clearly shows that an appropriate oxide thickness (0.16 nm) is crucial for obtaining a strongest LPE in MOS structure. In fact, the oxide layer in this case is less than one monolayer, which means the oxide molecules cannot fully cover or dust the semiconductor substrate. The mechanism will be discussed in **3.6**.

### 2.6. The Influence of Laser Power and Wavelength on LPE

**Figure 8.**
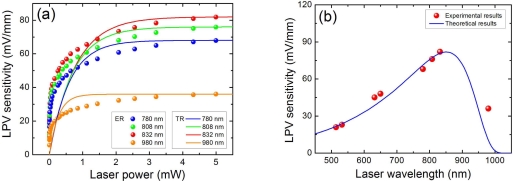
**(a)** LPV measurement in MS structure of Co(3.5 nm)/Si as a function of laser power with different light wavelength, where “ER” and “TR” represent the experimental results and theoretical results, respectively. **(b)** LPV measurement in MS structure of Co(3.5 nm)/Si as a function of light wavelength in Co(3.5 nm)/Si structure, where the light power is 5 mW. Here the contacts’ distance is 3.2 mm. Solid lines are the plots of Equation (23) and Equation (24), where the parameters are chosen as *E_g_* = 1.12 (eV), *d*_0_ = 2.9 (nm), *α* = 0.5, *β* = 2 and *P* = 0.5.

It was found that laser power and wavelength have an influence on the LPE [46]. Figure 8(a) shows the LPE as a function of light power at different wavelengths in Co(3.5 nm)/Si structure. It is clear that, for each wavelength, the LPV sensitivities are always proportional to the light power as the applied power is low, and then slowly become saturated as the power is increased. For different light wavelength, the saturate value is different.

To better understand the LPE responding to light wavelength, we further measured the LPE as a function of light wavelength at a fixed light power of 5 mW. As shown in Figure 8(b), we find there exists an optimum wavelength that can produce the largest LPE. For Co(3.5 nm)/Si structure, the optimum wavelength is at 832 nm. In fact, this optimum wavelength can be modulated by the metal thickness in MS structure [46].

### 2.7. Contacts' Distance Effect

**Figure 9.**
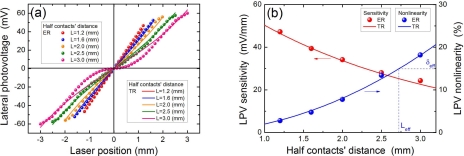
**(a)** LPV measurement in MS structure of Ti(6.2 nm)/Si with different contacts’ distance. Solid lines are the plots of Equation (8), where the parameters are chosen as *λ_m_* = 2.5 (nm). **(b)** LPV sensitivity and nonlinearity in MS structure of Co(3.5 nm)/Si as a function of half contacts’ distance. Solid lines are the plots of Equation (12) and Equation (14), where the parameters are chosen as *λ_m_* = 2.5 (nm). Here “ER” and “TR” represent the experimental results and theoretical results, respectively. The laser wavelength and power are 632 nm and 3 mW.

As we have mentioned in the Introduction, the two main criteria for LPE are the LPV sensitivity and LPV linearity. To obtain a perfect LPE, a large sensitivity with a small nonlinearity is necessary. In fact, these two criteria strongly depend on the contacts’ distance [50]. Figure 9(a) shows LPE in MS structure of Ti(6.2 nm)/Si with different contacts’ distance. Clearly, the increase in the contacts’ distance will decrease the linearity of LPV. As shown in Figure 9(b), when the contacts’ distance is less than 5.0 mm, the nonlinearity will be less than the acceptable value of 15% [64]. But when the contact’s distance becomes larger, the nonlinearity will accordingly become (e.g., 18.2% for 6.0 mm) larger than 15%. If we define an effective contacts’ distance, within which the LPV nonlinearity is less than the acceptable value, in this case, it should be 5.0 mm.

Figure 9(b) shows the increase in the contacts’ distance will decrease the LPV sensitivity. For example, when the contacts’ distance is at 2.4 mm, the LPV sensitivity can obtain a large value of 47.2 mV/mm, but when the contacts’ distance is extended to 6.0 mm, the LPV sensitivity will be decreased to a small value of 24.3 mV/mm. Therefore, a smaller contacts’ distance can not only achieve a larger LPV sensitivity but also obtain a higher linearity.

### 2.8. Vertical Offset Effect

**Figure 10.**
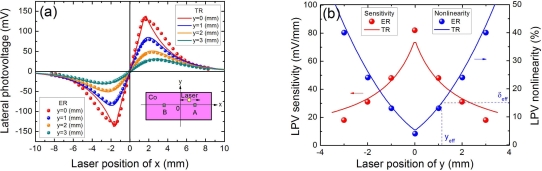
**(a)** LPV measurement in MS structure of Co(3.5 nm)/Si with different vertical distance of y. Solid lines are the plots of Equation (26), where the parameters are chosen as *λ_m_* = 2.5 (mm). **(b)** LPV sensitivity and nonlinearity in MS structure of Co(3.5 nm)/Si as a function of vertical distance of y. Solid lines are the plots of Equation (27) and Equation (28), where the parameters are chosen as *λ_m_* = 2.5 (nm). Here “ER” and “TR” represent the experimental results and theoretical results, respectively. The contacts’ distance is 3.2 mm, and the laser wavelength and power are 832 nm and 5 mW.

All the above experiments are focus on the LPE measurement within the line between two contacts. Figure 10(a) shows the LPE in Co(3.5 nm)/Si structure with different vertical distance of *y*. Please note all the LPVs with different *y* are measured in *x* direction. We can see that, when the vertical distance is increased, the linearity of LPV will be gradually decreased. As shown in Figure 10(b), when the vertical distance is 3.0 mm, the nonlinearity will become 20.1% which is larger than the acceptable value 15%. Similarly to the effective contacts’ distance discussed in **2.7**, if we define an effective vertical distance as a y position at which the LPV nonlinearity is less than the acceptable value, in this case, it should be 2.0 mm. Further, from Figure 10(b), the increase in the vertical distance will decrease the LPV sensitivity. Therefore, a smaller vertical offset can achieve not only a larger LPV sensitivity but also a higher linearity.

## 3. Theoretical Models

### 3.1. Electron Transition

**Figure 11.**
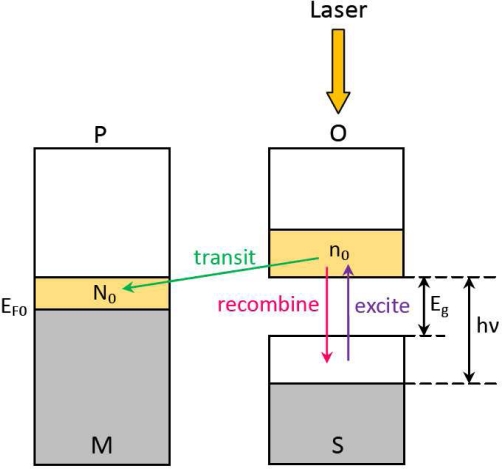
Diagram of electron transition from semiconductor to metal induced by laser illumination. The gray parts and the yellow parts represent the original equilibrium electrons and the laser-induced non-equilibrium electrons, respectively.

To explain the mechanism behind the LPE in MS (or MOS) structure, we propose the following model. As shown in Figure 11, when a laser with frequency *ν* and power *p* illuminates on the MS (or MOS) structure, electron-hole pairs are generated inside the semiconductor at light position. According to the absorption theory [67], the density of light-excited electrons can be written as:

(2) $$n_{0}=K{\left(\mathrm{hv}-E_{g}\right)}^{\alpha }$$

where *E_g_* is the energy gap of the semiconductor, *K* is a proportional coefficient, and *α* is an exponential coefficient.

These excited electrons will thus have a possibility (1 – *P*) to transit from the semiconductor into the metallic film through the Schottky barrier due to the non-equilibrium state, and meanwhile have a possibility (*_P_*) to recombine with the holes [46], as shown in Figure 11. Please note, here 0 ≤ *P* ≤ 1 is related with the Schottky barrier height. Generally, a laser with a larger power will result in more electrons transiting from semiconductor to metal because the recombined electrons have more opportunity to be re-excited by photons. Statistically, each electron among *n*_0_ can be re-excited *τp/n*_0_ times averagely, where *τ* is a time-related coefficient. Thus the transition electrons from semiconductor to metal at light position can be written as:

(3) $$N_{0}=n_{0}[1-P^{\left(\tau p/n0\right)}]$$

### 3.2. Carrier Diffusion

**Figure 12.**
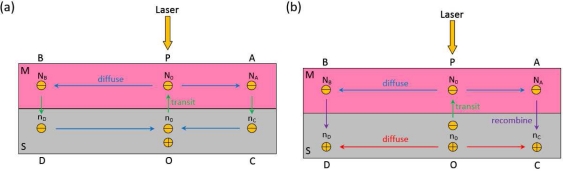
Diagram of carrier diffusion in MS structure induced by laser illumination. **(a)** Pure-electron picture of diffusion model. **(b)** Electron-hole picture of diffusion model.

**Figure 13.**
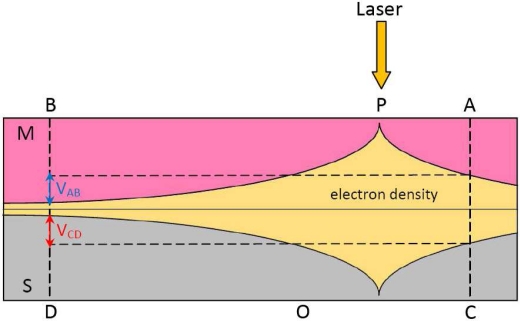
Diagram of laser-induced stable distribution of electron density in MS structure. The yellow part represents the distribution of density of laser-induced electrons.

Based on above discussion, the light-induced excess electrons in metal will thus generate a gradient laterally between the illuminated and the non-illuminated zones, resulting in excess electrons diffusing laterally along the metal away from the illuminated spot (at position P) toward two sides (at position A and B), as shown in Figure 12(a). To finish a circulation, these light-induced electrons in the metal will transit back to the semiconductor at non-illumination zones (at position C and D), and then go back to their starting spot (at position O). If a light keeps illuminating, the circulation will continue and a stable distribution of density of light-induced electrons will be kept, as shown in Figure 13.

The above discussion involves only the diffusion of electrons, and the diffusion of holes in semiconductor can be treated as the “electron diffusion” in an inverse direction. In fact, this diffusion model can also be interpreted by view of electron-hole different from above view of only electron being involved. Based on this view, as shown in Figure 12(b), the light-induced excess electrons in metal and holes in semiconductor will thus generate a gradient laterally between the illuminated and the non-illuminated zones, resulting in excess electrons (or holes) diffusing laterally along the metal□(or semiconductor) away from the illuminated spot. To finish a circulation, these light-induced electrons in the metal and holes in semiconductor will recombine at non-illumination zones. Please note, the carrier’s drift in the structure caused by the diffusion potential due to carriers’ diffusion can be neglected because of the low diffusion potential (regarded as the lateral photovoltage).

According to diffusion equation of $D_{m}\frac{d^{2}N(r)}{dr^{2}}=\frac{N(r)}{\tau_{m}}$, the density of electrons in the metal at position *_r_* can be written as:

(4) $$N(r)=N_{0}\;\text{exp}(-\frac{\left|x-r\right|}{\lambda_{m}})$$

here *x* is the laser spot position. $D_{m}=\frac{k_{B}T}{N_{F0}e^{2}\rho }$ is the diffusion constant of the metal, where $N_{F0}=\frac{8\pi }{3}{\left(\frac{2m_{e}E_{F0}}{\hbar^{2}}\right)}^{\frac{3}{2}}$ is the electron density below Fermi level of *E_F_*_0_ at equilibrium state in the metal and *ρ* is the resistivity of the metal. *τ_m_* is the life time of diffusion electrons in the metal. $\lambda_{m}=\sqrt{D_{m}\tau_{m}}$ is the electron diffusion length in the metal. Please note, here we only consider the one-dimensional situation in *x* direction, and the two-dimensional situation will be discussed in Section 3.9.

Similarly, if we suppose the electron diffusion length in the semiconductor to be $\lambda_{s}=\sqrt{D_{s}\tau_{s}}$ where *D*_s_ is the diffusion constant of the semiconductor and *τ_s_* is the life time of diffusion electrons in the semiconductor, then the density of diffusion electrons in the semiconductor at position *r* can be written as:

(5) $$n(r)=n_{0}\;\text{exp}(-\frac{\left|x-r\right|}{\lambda_{s}})$$

Equation (4) and Equation (5) tell us that the electron density in metal and semiconductor will both form an exponential distribution, as shown in Figure 13.

### 3.3. Energy Band Profile

**Figure 14.**
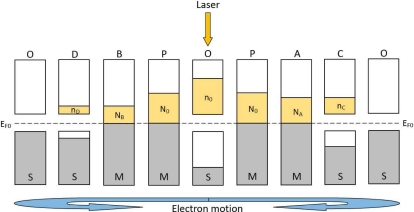
Diagram of energy band in MS structure illuminated by a laser. The gray parts and the yellow parts represent the original equilibrium electrons and the laser-induced non-equilibrium electrons, respectively.

Figure 14 shows the schematic energy band profile in MS structure illuminated by a laser. Due to the increase in the electron density induced by laser-illumination, the Fermi level in both the metal and the semiconductor will be increased. Thus, the new Fermi level in the metal and the semiconductor at position *r* can be respectively calculated as:

(6) $$E_{\mathrm{Fm}}(r)=E_{F0}+\frac{1}{4\pi }{\left(\frac{\hbar^{2}}{2m_{e}}\right)}^{\frac{3}{2}}{E_{F0}}^{-\frac{1}{2}}N(r)$$

(7) $$E_{\mathrm{Fs}}(r)=E_{F0}+\frac{k_{B}T}{n_{T}}n(r)$$

where $n_{T}=\frac{2{\left(2\pi m_{e}k_{B}T\right)}^{\frac{3}{2}}}{h^{3}}\text{exp}(-\frac{E_{c}-E_{F0}}{k_{B}T})$ is the electron density in conduction band of the semiconductor due to temperature fluctuation.

If the lateral distance of the laser spot from each contact is different, then the electron density at two contacts is different. This will result in the difference in the Fermi level at two contacts and thus generates a LPV. The LPV in the metal side and semiconductor side can be respectively presented as:

(8) $$V_{m}\equiv V_{\mathrm{AB}}=\frac{E_{\mathrm{Fm}}(L)-E_{\mathrm{Fm}}(-L)}{e}=K_{m}N_{0}[\text{exp}(-\frac{\left|L-x\right|}{\lambda_{m}})-\text{exp}(-\frac{\left|L+x\right|}{\lambda_{m}})]$$

(9) $$V_{s}\equiv V_{\mathrm{CD}}=\frac{E_{\mathrm{Fs}}(L)-E_{\mathrm{Fs}}(-L)}{e}=K_{s}n_{0}[\text{exp}(-\frac{\left|L-x\right|}{\lambda_{s}})-\text{exp}(-\frac{\left|L+x\right|}{\lambda_{s}})]$$

here *L* and –*L* are the positions of two contacts. $K_{m}=\frac{1}{4\pi e}{\left(\frac{\hbar^{2}}{2m_{e}}\right)}^{\frac{3}{2}}E_{F0}^{\;-\frac{1}{2}}$ and $K_{s}=\frac{k_{B}T}{{\mathrm{en}}_{T}}$ are two proportional coefficients. If we suppose *L* << *λ_m_* and *L* << *λ_s_* [50], then the LPV (according to Equation (8) and Equation (9)) by the laser position of *x* within [−*L*, *L*] can be idealized as:

(10) $$V_{m}^{i}=\frac{2K_{m}N_{0}}{\lambda_{m}}\text{exp}(-\frac{L}{\lambda_{m}})x$$

(11) $$V_{s}^{i}=\frac{2K_{s}n_{0}}{\lambda_{s}}\text{exp}(-\frac{L}{\lambda_{s}})x$$

Clearly, Equation (10) and Equation (11) give the linear relationship between LPV and laser position, which is the most significant characteristic of LPE.

### 3.4 LPV Sensitivity and Nonlinearity

According to Equation (10) and Equation (11), the LPV sensitivity in metal side and semiconductor side can be respectively written as:

(12) $$\kappa_{m}=\frac{2K_{m}N_{0}}{\lambda_{m}}\text{exp}(-\frac{L}{\lambda_{m}})$$

(13) $$\kappa_{s}=\frac{2K_{s}n_{0}}{\lambda_{s}}\text{exp}(-\frac{L}{\lambda_{s}})$$

It has been clear that the LPV sensitivity relates with the physical properties of both metal and semiconductor materials, and this will result in many effects of LPE, such as thickness effect and so on. This will be discussed later.

According to Equation (1), the LPV nonlinearity in metal side and semiconductor side can be calculated as:

(14) $$\delta_{m}=\frac{2\sqrt{\{\int_{-L}^{L}{\left[V_{m}(x)-V_{m}^{i}(x)\right]}^{2}dx\}/2L}}{V_{m}^{i}(L)}=\frac{1}{3\sqrt{7}}{\left(\frac{L}{\lambda_{m}}\right)}^{2}$$

(15) $$\delta_{s}=\frac{2\sqrt{\{\int_{-L}^{L}{\left[V_{s}(x)-V_{s}^{i}(x)\right]}^{2}dx\}/2L}}{V_{s}^{i}(L)}=\frac{1}{3\sqrt{7}}{\left(\frac{L}{\lambda_{s}}\right)}^{2}$$

If *L* << *λ_m_* and *L* << *λ_s_*, then the nonlinearity will become very small. This means the LPV will show a very linear characteristic response to the laser position. This is consistent with the condition of Equation (10) and Equation (11).

### 3.5. Metal Thickness Effect

**Figure 15.**
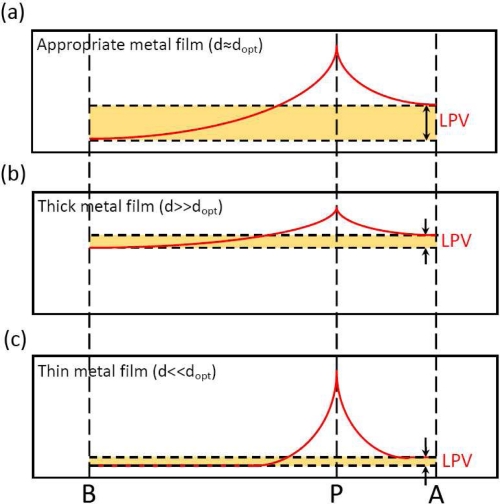
Diagram of metal thickness effect of LPE in MS structure. The red curves represent the distribution of density of laser-induced electrons. **(a–c)** shows the situations with three different metal thickness.

We have discussed in Section 2.4 (see Figure 5) that the LPE in MS structure has a great bearing on the metal thickness. In order to obtain the largest LPV sensitivity, an optimum metal thickness is crucial. The mechanism behind this metal thickness effect can be interpreted by Figure 15. If the metal film is very thick, as shown in Figure 15(b), then the electrons can easily diffuse from the light spot position toward two contacts because of the small resistivity, thus the density of electrons at two contacts are both high, resulting in a small difference of metallic potential between them (see yellow part), *i.e.*, a small LPV. Similarly, if the metal film is very thin, as shown in Figure 15(c), then the electrons can hardly diffuse because of the large resistivity, thus the density of electrons at two contacts are both low, also resulting a small difference of metallic potential between them, ie a small LPV. Therefore, in order to obtain a large difference of metallic potential between two contacts, an appropriate metal thickness is needed, as illustrated in Figure 15(a).

Equation (12) has told that the LPV sensitivity is closely linked with the electron diffusion length. According to Ref. [50], for a very thin metal film, the electron diffusion length has a linear relationship with the metal thickness, which can be presented as:

(16) $$\lambda_{m}=\alpha_{m}(d-d_{0})$$

where *d* is the metal thickness, *α_m_* is a proportional coefficient, and is the threshold thickness. Thus substituting Equation (16) into Equation (12), the LPV sensitivity can be written as:

(17) $$\kappa_{m}(d)=\frac{2K_{m}N_{0}}{\alpha_{m}(d-d_{0})}\text{exp}[-\frac{L}{\alpha_{m}(d-d_{0})}]$$

The theoretical result according to Equation (17) is well consistent with the experimental results, as shown in Figure 5(b).

### 3.6. Oxide Thickness Effect

As that interpreted in Section 2.5, the LPE in MOS structure is closely related with the oxide thickness. Due to the existence of oxide layer which serves as a relatively high barrier, the LPE in MOS structure always shows a smaller LPV sensitivity than that in MS structure [as shown in Figure (6)]. With the increase in the oxide thickness, the LPV sensitivity will decrease. However, as shown in Figure 7, when the oxide thickness becomes very thin, the LPE in MOS structure shows a larger LPV sensitivity than that in MS structure. Besides, there also exists an optimum thickness at which the LPV sensitivity has the largest value.

**Figure 16.**
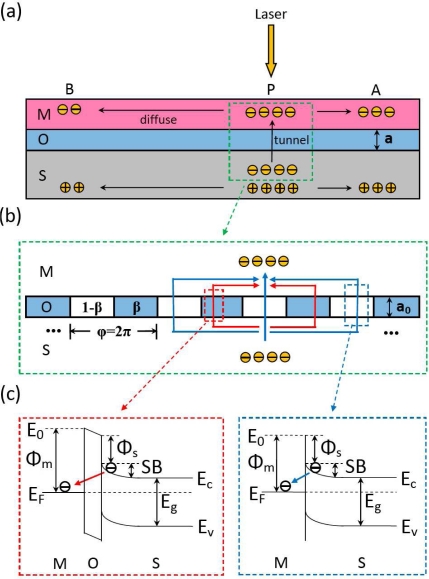
Diagram of oxide thickness effect of LPE in MOS structure. **(a)** LPE mechanism in MOS structure with a fully covered oxide layer. **(b)** Electronic interference based on un-fully covered oxide layer when excited electrons transit from semiconductor to metal. The red (or blue) arrows represent the electrons tunneling through the walls (or passing through the windows) from semiconductor to metal. **(c)** Band models of electrons as tunneling through the walls (red part) and passing through the windows (blue part) from semiconductor to metal.

In order to explore the mechanism behind this oxide thickness effect, we give the following explanation based on electrons interference, as shown in Figure 16. Generally, the oxide layer at interface always decreases the possibility of the tunneling of electrons from semiconductor to metal, thus deteriorates the formation of LPV in MOS structure, as shown in Figure 16(a). However, when the thickness of an oxide layer becomes very thin and even less than one monolayer, the oxide molecules cannot fully cover or dust the semiconductor substrate, as shown in Figure 16(b). In this case, the tunneling behavior of electrons will become quite different compared with that in a fully covered MOS structures. Thus we define a $\beta =\frac{a}{a_{0}}$ (*a* < *a*_0_) to describe this situation, where *α* is the nominal thickness of oxide layer and *α*_0_ is the thickness of one complete monolayer. So there will be *β*% of the region [hereafter we call it the “wall”, see Figure 16(b)] that is occupied by the oxide molecules and another part of (1 − *β*)% that is empty (hereafter we call it the “window”). Thus, when electrons transit from semiconductor to metal near the light position, they can either pass through the “windows” directly or tunnel through the “walls”, as shown in Figure 16(c). Therefore, as shown in Figure 16(b), the final wavefunction of electrons after superposition at light position can be written as:

(18) $$\Psi =\{\begin{array}{ll} \sum_{1-\beta }\phi (r)+\sum_{\beta }\phi (r)e^{-\frac{a_{0}}{a_{t}}} & \left(0<a<a_{0}\right)\\ \int_{r}\phi (r)e^{-\frac{a}{a_{t}}}dr & \;(a\geq a_{0}) \end{array}$$

here *α_t_* is the electron tunneling length in the oxide layer, and $\phi (r)=\phi_{0}(r)\times e^{i\varphi (r)}={\left(\frac{n_{0}e^{-r/2\lambda_{s}}}{2\lambda_{s}}\right)}^{\frac{1}{2}}\times e^{i2\pi \frac{P_{e}}{h}r}$ is sub-wavefunction passing through oxide layer at lateral position *r* from light position, where *ϕ*_0_(*r*) is the amplitude and *φ*(*r*) is the phase, *n*_0_ is the density of light-excited electrons at light position in the semiconductor, *λ*_s_ is the electron diffusion length in semiconductor, and *p_e_* is the average momentum of transiting electrons. Thus the density of light-excited electrons at light position in the metal after tunneling can be written as:

(19) $$N_{0}=\Psi^{*}\Psi$$

Because the metal side LPV is proportional to the electron density at light position, which can be presented as *V_AB_* = *K_m_N*_0_, thus the LPV can be calculated as:

(20) $$V_{\mathrm{AB}}=\{\begin{array}{ll} A\times {\left[B^{2}+C^{2}e^{-2D(1-\beta )}-2\mathrm{BC}e^{-D(1-\beta )}\text{cos}(2\pi \beta )\right]}^{2} & \left(0<a<a_{0}\right)\\ A{\left(1-C\right)}^{3}\times {\left(1-e^{-D}\right)}^{4}e^{-a/a_{t}} & \left(a\geq a_{0}\right) \end{array}$$

here $A=K_{m}n_{0}{\left[\frac{2\lambda_{s}D^{2}}{4\lambda_{s}^{2}+D^{2}}\right]}^{2}{\left[\frac{1+\text{exp}(-D)}{1-\text{exp}(-D)}\right]}^{4}$, *B* = 1−*e*^−*a*_0_/*a*_*t*_−*D*^, *C* = 1−*e*^−*a*_0_/*a_t_*^, and $D=\frac{1}{4p_{e}\lambda_{s}}$. We can see from Figure 6(b) and Figure 7(b) that the theoretical results according to Equation (20) is well consistent with the experimental results.

It is interesting why an un-fully covered oxide layer at interface of MOS structure can cause such a huge LPV. This can be explained from the viewpoint of electrons interference. In fact, as we have mentioned above, the thickness change of an un-fully covered layer modulates the occupancy *β* of oxide molecules, which greatly affects the superposition of all sub-wavefunctions at light position in metal. For a proper *β* (a crucial parameter that determines the scale of “walls” and “windows” which are considered to array periodically), all the sub-wavefunctions with positive phase (defined as (2*kπ*, 2*kπ* + *π*), *k =* 0,1,2···) can directly pass through 1 − *β* “windows” and all the sub-wavefunctions with negative phase (defined as (2*kπ* + *π*, 2*kπ* + 2*π*), *k =* 0,1,2···), will be obstructed by *β* “walls”, resulting in an enhancement of interference, as shown in Figure 16(b). This quite likes the phenomenon of interference enhancement as a light goes through a Fresnel zone plate.

### 3.7. Selection of Materials

The properties of the metal and semiconductor materials play an important role on the metal side LPE in MS structure. The selection of an appropriate metal material or a suitable semiconductor material is important for obtaining a large LPV sensitivity and a good linearity. Substituting Equation (2), Equation (3) and the expression of *λ_m_* into Equation (12) and Equation (14), the LPV sensitivity and nonlinearity can be obtained as:

(21) $$\kappa_{m}={\left[K{\left(\frac{\hbar^{2}}{2m_{e}}\right)}^{\frac{3}{4}}{\left(\frac{2}{3\pi k_{B}T}\right)}^{\frac{1}{2}}\right]}_{C}\times {\left[{\left(\mathrm{hv}-E_{g}\right)}^{\alpha }\right]}_{S}\times {\left[E_{F0}^{\;\frac{1}{4}}\rho^{\frac{1}{2}}\tau_{m}^{\;-\frac{1}{2}}\right]}_{M}$$

(22) $$\delta_{m}={\left[\frac{8\pi e^{2}L^{2}}{9\sqrt{7}k_{B}T}{\left(\frac{2m_{e}}{\hbar^{2}}\right)}^{\frac{3}{2}}\right]}_{C}\times {\left[E_{F0}^{\;\frac{3}{2}}\rho \tau_{m}^{-1}\right]}_{M}$$

here we have supposed the contacts’ distance is small enough (*L* << *λ_m_*) and the laser power is strong enough ( $p>>\frac{n_{0}}{\tau }$). The coefficients in the square brackets with a subscript of “C”, “S” and “M” represent the “constant”, “semiconductor-related” and “metal-related” coefficients, respectively.

**Figure 17.**
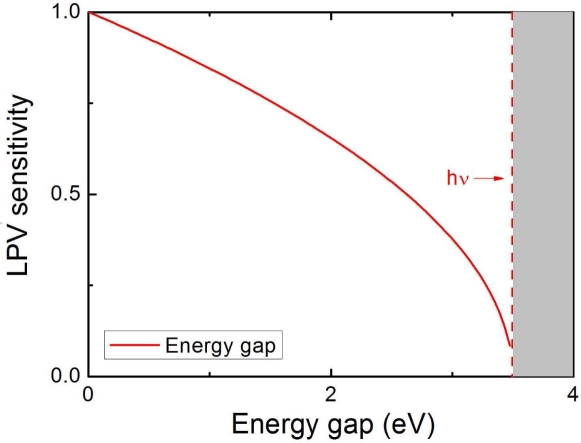
Calculation of normalized LPV sensitivity responding to the energy gap of semiconductor materials in MS structure according to Equation (21), where the parameters are chosen as *ν* = 3.5 (eV)/*h* and *α* = 0.5.

Figure 17 shows the metal side LPV sensitivity responding to the energy gap of semiconductor materials. Obviously, the semiconductors with a smaller energy gap can often obtain a larger LPV sensitivity. This is because, with a small energy gap, more electrons, which participate in the LPV formation, can be excited from valence band to conduction band in the semiconductor. It must be stressed here that the nature of semiconductor materials doesn’t affect the LPV nonlinearity in metal side.

**Figure 18.**
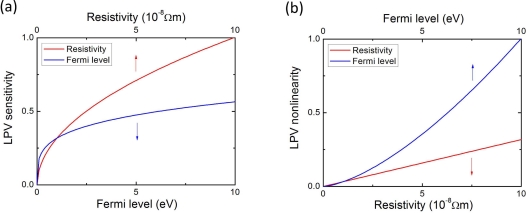
Calculation of normalized **(a)** LPV sensitivity and **(b)** nonlinearity responding to metal Fermi level and metal resistivity in MS structure according to Equation (21) and Equation (22).

Figure 18 shows the LPV sensitivity and nonlinearity responding to the Fermi level and resistivity of metal materials. Here we have supposed the *τ_m_* keeps unchanged for different metal materials. The results show that the resistivity and the Fermi level are the two crucial factors for metal side LPE. The metal with higher resistivity and higher Fermi level can produce a higher LPV sensitivity, but results in a larger nonlinearity. In order to obtain a large LPV sensitivity with a small nonlieanrity, choosing a metal material with appropriate Fermi level and resistivity is necessary.

### 3.8. The Influence of Laser Power and Wavelength on LPE

We have discussed in experiments in Section 2.6 that the LPV sensitivity in MS structure has a relationship with the laser power as well as its wavelength. A laser with large power can easily saturate the sensitivity, while the optimum wavelength is also needed for obtaining the largest sensitivity. Substituting Equation (3) into Equation (12), we get:

(23) $$\kappa_{m}(p)=\frac{2K_{m}n_{0}e^{-L/\lambda_{m}}}{\lambda_{m}}[1-P^{\left(\tau p/n_{0}\right)}]$$

According to Equation (23), if the light power is large enough, then the LPV sensitivity will be saturated. This is consistent with the above experimental results [see Figure 8(a)].

It is easy to understand that the electrons with a larger rest energy of (*hv* − *E_g_*) after transition in semiconductor possess a longer diffusion length in metal, which can be written as *λ_m_* = *K*′(*hv* – *E_g_*)^β^, where *K*′ is a proportional coefficient and *β* is an exponential coefficient. Thus substituting Equation (2) into Equation (23) and supposing the light power is large enough, then the LPV sensitivity can be written as:

(24) $$\kappa_{m}(v)=\frac{2{\mathrm{KK}}_{m}}{K\prime }{\left(\mathrm{hv}-E_{g}\right)}^{\alpha -\beta }\text{exp}[-\frac{L}{K\prime {\left(\mathrm{hv}-E_{g}\right)}^{\beta }}]$$

Figure 8(b) shows a comparison of theoretical results and experimental results of LPV sensitivity *vs.* laser wavelength, and we find they are consistent well with each other.

### 3.9. Effective Linear Area

We have seen in Section 2.7 that the LPV linearity depends on two contacts’ distance. When the contacts’ distance is small, the LPV shows a good linearity *vs.* laser position, but when the contacts’ distance becomes large, the linearity will be decreased, as shown in Figure 9. This result can also be interpreted by the theoretical model. Equation (8) gives the relationship between LPV and laser position, and we can see that they are not proportional with each other. Only when the condition of *L* << *λ_m_* is satisfied, a linear characteristic can be achieved, as shown in Equation (10). This is why a larger contacts’ distance will deteriorate the linearity.

**Figure 19.**
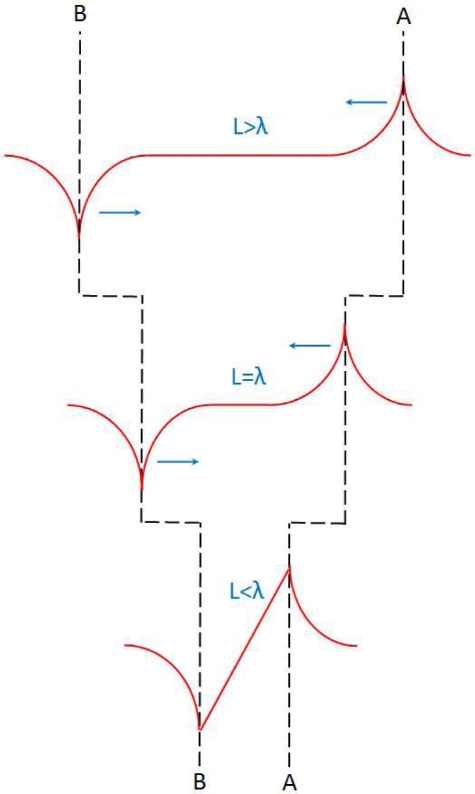
Diagram of LPV responding to laser position (see red curves) with different contacts’ distance.

Figure 19 gives a simple picture regarding the contacts’ distance effect. When two contacts’ distance is very large (compared with the diffusion length), electron density at one contact (which is close to the laser spot) can only be affected by the change of laser position, and the electron density at other end cannot be affected because this contact point is far away from the laser spot (exceeds the diffusion length).

So the LPV, *i.e.*, the difference of electron density between two contacts, is only related with the electron density at one contact. Thus the LPV only shows an exponential relationship with laser position (according to Equation (4) and Equation (6)). But when the contacts’ distance becomes small (compared with the diffusion length), the electron density at two contacts can be both affected by the change of laser position. Therefore, in this case, two exponential relationships should both be considered, and according to the Taylor expansion, the zero and the second order factors will be eliminated by subtraction between them, and only the first (*i.e.*, the linear) order factor will be kept. Thus the LPV shows a linear relationship with laser position. As discussed in Section **2.7** [see Figure 9(b)], if we define an effective contacts’ distance as a region within which the LPV nonlinearity is less than the acceptable value, then according to Equation (14), this effective contacts’ distance can be written as:

(25) $$2L_{\mathrm{eff}}={\left[12\sqrt{7}\delta_{\mathrm{eff}}\right]}^{\frac{1}{2}}\lambda_{m}$$

where *δ_eff_* is the acceptable value of nonlinearity.

We also know from **2.8** that the increase in the vertical distance of *y* will both decrease the LPV sensitivity and linearity, as shown in Figure 10(b). In fact, if the vertical distance is not zero, the Equation (8) should be written as:

(26) $$V_{\mathrm{AB}}=K_{m}N_{0}\{\text{exp}[-\frac{\sqrt{{\left(L-x\right)}^{2}+y^{2}}}{\lambda_{m}}]-\text{exp}[-\frac{\sqrt{{\left(L+x\right)}^{2}+y^{2}}}{\lambda_{m}}]\}$$

Based on Equation (26), the LPV sensitivity and the nonlinearity [see Equation (12) and Equation (14)] can be recalculated as:

(27) $$\kappa_{m}(y)=\frac{2K_{m}N_{0}}{\lambda_{m}}\text{exp}(-\frac{L}{\lambda_{m}})\times (\frac{L}{L+y})$$

(28) $$\delta_{m}(y)=\frac{1}{3\sqrt{7}}{\left(\frac{L+y}{\lambda_{m}}\right)}^{2}$$

**Figure 20.**
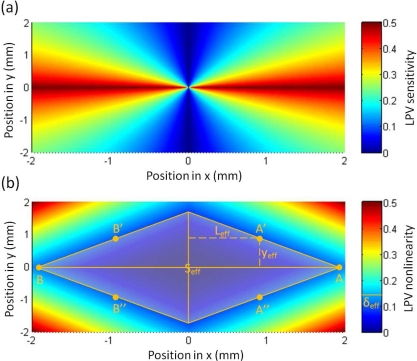
Calculation of normalized **(a)** LPV sensitivity and **(b)** nonlinearity *vs.* both contacts’ distance and vertical offset distance according to Equation (27) and Equation (28), where the parameters are chosen as *λ_m_* = 2 (mm).

Figure 20 shows the LPV sensitivity and nonlinearity responding to both contacts’ distance and vertical offset distance. A larger sensitivity with a smaller nonlinearity can be obtained in case of smaller contacts’ distance and smaller vertical offset distance. This is why we always put laser spot at the line between two contacts and minimize two contacts’ distance as small as possible. Similarly to the definition of effective contacts’ distance, we can define an effective vertical offset distance of *y_eff_* [see Figure 10(b)], at which the LPV nonlinearity is less than the acceptable value of *δ_eff_*, according to Equation (28), this effective vertical offset distance can be written as:

(29) $$y_{\mathrm{eff}}={\left[3\sqrt{7}\delta_{\mathrm{eff}}\right]}^{\frac{1}{2}}\lambda_{m}-L_{\mathrm{eff}}$$

Equation (29) indicates, to keep the nonlinearity within an acceptable range, any increase in the vertical offset distance should be compensated by the decrease in the effective contacts’ distance. Therefore, we can define an effective linear area (in the surface of the device), within which the LPV nonlinearity is less than the acceptable value of *δ_eff_*. According to Equation (29), this effective linear area [see Figure 20(b)] can be calculated as:

(30) $$S_{\mathrm{eff}}=6\sqrt{7}\delta_{\mathrm{eff}}\lambda_{m}^{2}$$

## 4. Conclusions

This review deals with some of our recent works on LPE in MS and MOS structures. Large LPVs with good linearities were achieved in these structures. Some important factors which greatly affect the LPE were analyzed, such as thickness effect, contacts’ distance effect, vertical offset effect, material selection, and influence of laser power and wavelength. A concise model regarding LPE in MS and MOS structures was also given, showing a good consistency with the experimental observations. Moreover, our recent studies show these structures can also present a novel bipolar-resistance effect (BRE) [68,69], indicating MS (MOS) structure a great potential for the development of future versatile photoelectric devices.
